# Aluminum—Its Mode of Working, and Alloys

**Published:** 1870-02

**Authors:** 


### Selections.

---

Aluminum—Its Mode of Working, and Alloys—*Soldering Aluminum.*—The peculiar difficulty which was encountered for years in the soldering of aluminum has been a great drawback for its more general application. The common method of brazing with borax is not applicable for this metal, because it corrodes and oxidizes it. At first, tin solder was used, but that afforded little solidity; and riveting was soon found out to be too tedious a process. Happily the difficulty has now been surmounted by Mouray, of Paris. The specimens of articles manufactured by his method were first exhibited at one of the meetings of the famous *Societe d'Encouragement.* Among these were especially noticed a coffeepot with eight solderings, several eagles for the banners of the French army, and a trumpet, consisting of forty-two parts.

Soldered strips of sheet aluminum in being bent to and fro, never gave way at the soldered spot, but always outward of the same, which, as is well known, is not the case with the best silver soldering.

Mouray employs five different solders, which are composed as follows:

| No. 1. | No. 2. | No. 3. | No. 4. | No. 5. | |
|---|---|---|---|---|---|
| 80 | 85 | 88 | 90 | 94 | parts in weight of zinc. |
| 8 | 6 | 5 | 4 | 2 | " " copper. |
| 12 | 9 | 7 | 6 | 4 | " " aluminum. |

These ingredients are melted in a crucible. The copper is fused first, and the aluminum is then added in three or four portions. When the whole is liquefied, it is stirred with an iron rod. The crucible is then withdrawn, and the zinc introduced into the mass under constant stirring. It should be free from iron. The liquefied mass is poured in ignot-like molds, which have been wiped out with benzine.

The selection of the solder depends upon the nature of the object. In order to quicken its fusion on the metal, a mixture of three parts of balsam of copaiva and one part venetian turpentine is made use of; otherwise the operation is performed in exactly the same manner as in the brazing of other metals. The aluminum solder is spread without delay on the previously heated surfaces to be fastened together. In heating, the blue gas flame or the turpentine blast lamp is employed. The more and oftener the solder is spread over the surface the better it is.

*On Other Manipulations in the Working of Aluminum.*—In order to avoid trouble in casting aluminum, the metal should not be put all at once in the crucible, but only in small portions, and new ones should not be added until those previously added are melted. Oxidation is prevented by previously dipping the pieces in benzole, and when it is intended to melt the dripping obtained in the working of this metal, it is necessary to clean them from the solder which may adhere to them, otherwise the casting will be spoiled. By allowing the pieces to remain for some time in nitric acid, the solder is corroded but the aluminum is left untouched.

The annealing of articles made of aluminum is not attended with more difficulties than that of other metals. The operation is performed when the metal commences to glow; in case, however, that fears should be entertained about the striking of the right moment, the object to be heated may be spread over with some fatty matter, which, in disappearing, indicates the time when the object has to be withdrawn from the furnace.

When to be rolled out, it must be annealed oftener than other metals. This is now attained with great ease.

In 1857, the cost of the rolling of one pound of aluminum amounted to 13 1-3 Prussian thalers, while at present it is only one tenth of that price. In burnishing or spinning aluminum in the lathe, it is necessary to make use of a varnish, consisting of four parts of turpentine and one of stearic acid:

One of the many interesting peculiarities of the new metal is its property of resisting the action of the graver, which slides off from its surface as if it were glass. When, however, a mixture of rum and the above-mentioned varnish is employed, the graver penetrates into it as if it were pure copper.

In polishing of aluminum, the substances generally employed for this operation are of no utility. Mouray recommends the use of an emulsion of equal parts of rum and olive oil, made by shaking these liquids together in a bottle. When the stone is used, the peculiar black streaks first appearing should not be a reason of vexation, since they do not injure the metal in the least, and may be removed with a woolen rag. The objects in question may also be brightened in potassa lye, in which case, however, care must be taken in not making use of too strong a lye. For cleaning purposes, benzole has been found best.

Finally, it may be mentioned that objects of aluminum can be electroplated without the least difficulty, and Mouray succeeded in imparting to them a bright, white luster in passing them successively through a weak bath of hydrofluoric acid and aqua-fortis. The effect thus attained is said to be really surprising.

*The Alloys of Aluminum.*—We have to distinguish between alloys in which the aluminum predominates and such ones in which the other metals outweigh the latter. Those impart to the aluminum new properties. Iron and copper do not act injuriously if the admixture is not considerable.

In regard to toughness, the union of seven per cent. of iron can scarcely be distinguished from pure aluminum. Both metals easily combine with each other. Commercial aluminum mostly contains iron; it remains ductile with as much as ten per cent. of copper, and when containing only half as much, it may be worked still easier. If alloyed with small quantities of zinc, tin, gold, or silver, the metal is rendered hard and more brilliant, but remains ductile. Especially recommended is the alloy consisting of ninety-seven per cent. of aluminum, and three per cent. of zinc. The alloy with seven per cent. of tin can be worked well, but does not take a very fine polish, and can not be cast, since a more fusible alloy with a large proportion of tin is separated.

Aluminum and lead do not unite. The composition with three per cent. of silver and ninety-seven of aluminum possesses a beautiful color, and in equal parts they yield an alloy of the hardness of bronze. The union of ninety-nine per cent. of aluminum and one of gold is, though hard, still ductile; its color is that of green gold. With ten per cent. of gold, the composition is rendered crystalline.

The most important alloy, however, is that composed of ninety per cent. of copper and ten per cent. of aluminum. It possesses a pale gold color, a hardness surpassing that of bronze, is susceptible of taking a fine polish, and is easier forged than soft iron. This alloy has found a ready market, and if less costly, would replace red and yellow brass. Its hardness and tenacity render it peculiarly adapted for journals and bearings.

Christofle, of Paris, who uses it for a journal for a polishing disk, found that it lasted six times longer than ordinary journals—that is, eighteen months. There were 2,200 revolutions made per minute. It is further stated, on good authority, that a journal of this new bronze which was employed for the axle of a sewing machine, making 240 revolutions per minute, did excellent service for one year without indicating the least deficiency. Journals of ordinary bronze do not, as is well known, last over five months.

